# Socio‐Economic Effects on the Temporal Importance of Breeding Site Types for *Aedes aegypti* in a Tropical Epidemic City

**DOI:** 10.1111/zph.70018

**Published:** 2025-10-07

**Authors:** Mariana Mayumi Zanoni, Luiz Gustavo Rodrigues Oliveira Santos, Alessandra Gutierrez de Oliveira

**Affiliations:** ^1^ Federal University of Mato Grosso Do Sul, Graduate Program in Infectious and Parasitic Diseases, Faculty of Medicine Campo Grande Brazil; ^2^ Federal University of Mato Grosso Do Sul, Graduate Program in Ecology, Institute of Biosciences Campo Grande Brazil

**Keywords:** arthropod vectors, entomology, insect control

## Abstract

**Introduction:**

*Aedes aegypti* (Diptera: Culicidae) is the vector of dengue, Zika, chikungunya, and yellow fever, arboviruses of major public health importance. The mosquito has a high adaptability, requiring the elimination of its primary breeding sites. In Brazil, breeding sites are classified by the Rapid Survey of Indices for *Aedes aegypti* (LIRAa) as water‐holding containers suitable for larval development. They are categorized into five groups: A (A1—elevated water tanks, A2—ground‐level water deposits), B (mobile containers), C (fixed containers), D (D1—tires, D2—trash), and E (natural breeding sites). This study aimed to verify whether the types of breeding sites changed in the course of 2 years and if socio‐economic factors, neighbourhood population density, and illiteracy rates impact the occurrence of these types of breeding sites.

**Method:**

Data were obtained from the larval surveillance program of the Vector‐borne Disease Control Coordination (CCEV) and socio‐economic data from the Brazilian Institute of Geography and Statistics (IBGE). Spatiotemporal variations were assessed using an Additive Multinomial Multilevel Statistical Model with a Bayesian approach. We hypothesized that areas with higher human population density would show a higher presence of mobile containers and trash, while areas with higher illiteracy rates would show a frequency of water tanks and trash. Regarding seasonality, we hypothesized that water‐filled water tanks and mobile containers would be more frequently present throughout the year.

**Results:**

Our findings highlight the predominance of trash and mobile containers, while natural breeding sites were the least relevant throughout the years analysed. Mobile containers' frequency increased in overcrowded neighbourhoods, and water tanks' frequency decreased. This finding suggests that urban density influences the frequency of these types of breeding sites. Areas with higher illiteracy rates showed a decrease in movable and fixed containers but an increase in trash and water tanks, indicating potential knowledge gaps or structural limitations in water storage practices.

**Conclusion:**

In conclusion, the variety of container types found in different urban and socioeconomic contexts emphasizes the need for interventions that are tailored to local conditions. These findings offer valuable insights for health agencies to improve entomological control strategies, potentially leading to a reduction in the incidence of arboviral diseases in Campo Grande, MS.

**Summary**

- Identification of key breeding sites
  ○ Trash and mobile containers are the primary breeding sites for *Aedes aegypti* . This insight can help improve mosquito control efforts by targeting the most significant sources of infestation.
- Socioeconomic impacts
  ○ Overcrowded areas have more mobile containers, while higher illiteracy regions show increased water tank breeding, linking social factors to mosquito proliferation.
- Enhancing vector control strategies
  ○ By understanding the link between breeding sites and socioeconomic factors, health authorities can create more effective interventions to reduce the spread of dengue, Zika, and other arboviruses.
- Impacts
  ○ We found that most *Aedes aegypti* mosquitoes, which spread dengue, Zika, and chikungunya viruses, come from trash like tires and bottles, not just big water tanks.
  ○ Every area has its own set of challenges. In neighbourhoods with a lot of people, mobile containers are the main problem, while in places with lower literacy, trash and water tanks are the most common breeding sites.
  ○ If we link breeding sites to the social conditions in a given area, health authorities can design targeted, community‐specific interventions. That makes campaigns against dengue, Zika, and chikungunya viruses more effective.

## 1 Introduction

The World Health Organization (WHO) categorised dengue as one of the top ten global public health threats, with an estimated 390 million infections occurring annually (WHO 2019). From the period of 1990–2021, dengue cases increased from 26.45 million to 58.96 million worldwide (Zhang et al. 2024). Approximately 90 countries have reported active dengue transmission, with more than 14 million cases and 11,000 dengue‐related deaths reported globally in 2024 (Venkatesan 2024; WHO 2024). Dengue prevention and containment efforts primarily rely on vector control; personal protective measures are recommended, such as vaccination that should be integrated into the control strategy. The recent availability of dengue vaccines represents an important advancement, but it does not replace vector control; the licensed vaccines are available only in specific locations and age groups due to issues related to efficacy and safety. Educational campaigns are also an important part of vector control for informing the population about the relevance of breeding sites and how to properly manage them (Sim et al. 2020; Paz‐Bailey et al. 2024). Nonetheless, the disease continues to spread quickly, both geographically and in terms of the frequency and severity of outbreaks (Murray et al. 2013).

*Aedes aegypti* Linnaeus, 1762 is the mosquito vector responsible for dengue virus transmission, and it thrives in urban environments where it finds various artificial breeding sites, such as containers with stagnant water (Valle et al. 2021). The adult females are hematophagous, meaning that they need blood meals for egg maturation; males generally feed on sugary substances, such as plant saps. Gravid females seek breeding sites for oviposition. The eggs are laid on these containers' walls and can remain dormant for over a year (Valle et al. 2021). When the eggs are submerged, the larvae emerge and undergo four distinct developmental stages, during which they feed on organic matter present. Following this period, the pupal stage ensues, from which the adults emerge (Nelson 1986).

**FIGURE 1:**
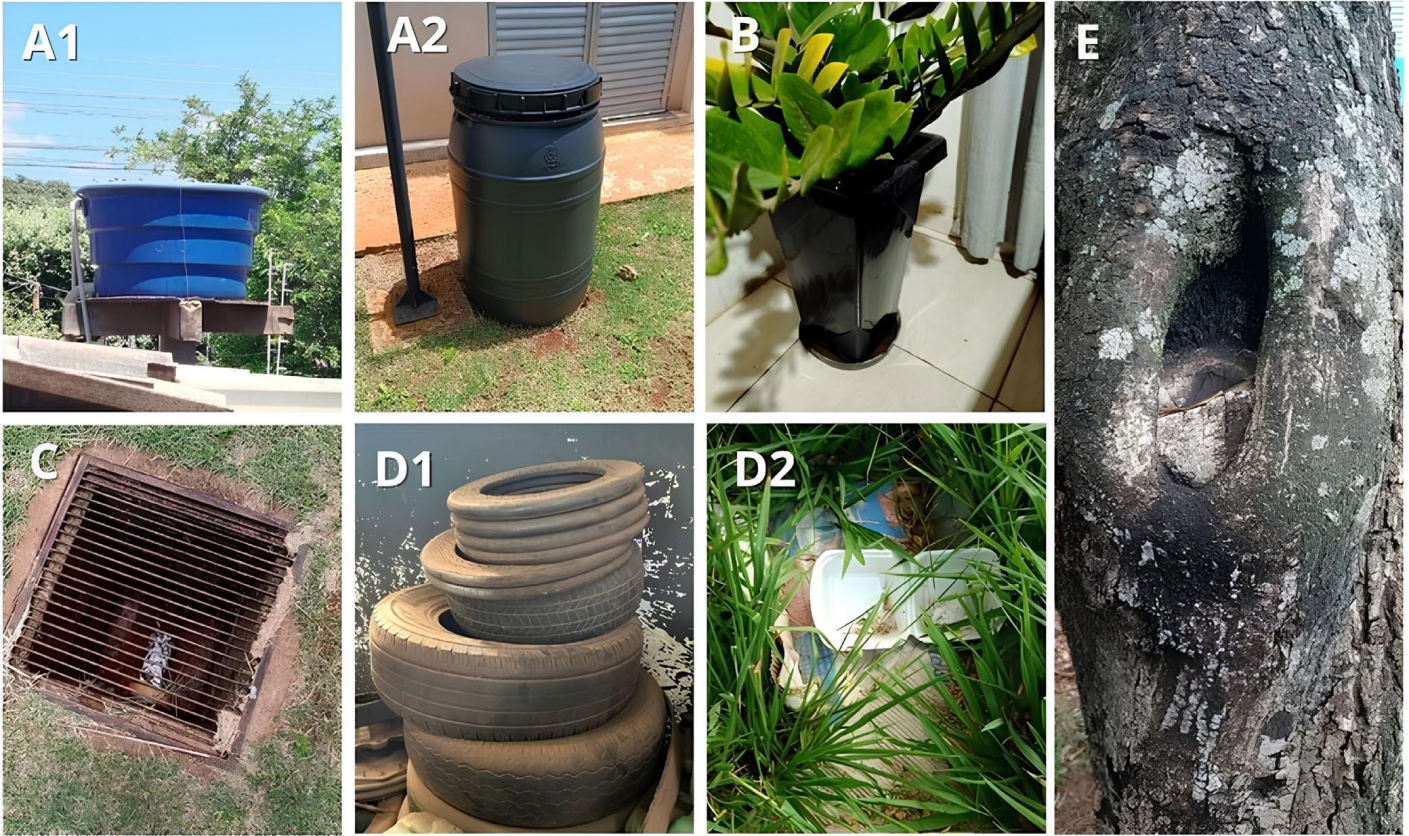
Breeding sites according to LIRAa. (A1) Water tanks, (A2) Ground‐level water deposits, (B) Mobile containers (e.g; plant pots and plates), (C) Fixed containers (e.g; drains), (D1) Tires, (D2) Trash, (E) Natural sites (e.g; tree hollow).

As a hematophagous mosquito, the *Ae. aegypti* female is a key public health threat as the vector of the etiological agents of dengue, Zika, chikungunya, and urban yellow fever. Due to its importance, entomological surveillance has been implemented to control this vector, focusing on both larval and adult stages. The key aspect during the larval stage is controlling breeding sites, as *Ae. aegypti* is known for its opportunistic behaviour in exploiting a wide range of them. Therefore, understanding which breeding sites are the most productive, where they are located, and what factors influence their abundance is a powerful tool for implementing targeted mosquito control (Wilke et al. 2019; Soares‐da‐Silva et al. 2012). In Brazil, the Rapid Assay of the Larval Index for *Aedes aegypti* (LIRAa) is a surveillance strategy to assess larval infestation of breeding sites, classified in five groups: A1 (elevated water tanks), A2 (ground‐level water deposits), B (mobile containers), C (fixed containers), D1 (tires), D2 (trash), and E (natural sites) (Figure 1). In this survey, the city was stratified into strata, and blocks within it were evaluated (Silva et al. 2020).

Campo Grande (Mato Grosso do Sul state, Brazil) is considered an epidemic center for dengue. In 2022, Campo Grande recorded 5467 cases, with an incidence rate of 603.4. Also in 2022, 17 deaths were reported in the state in July, six of them in Campo Grande (Brasil 2022). By December 2023, in the state of Mato Grosso do Sul, there were 40,887 confirmed cases with 42 deaths. In Campo Grande, 12,095 probable cases, with six deaths (Brasil 2023).

Areas with high human population density often result in inadequate housing, inefficient waste management, leading to the accumulation of garbage, including used tires, plastics, and cans, and a lack of public policies for monitoring arbovirus vectors (Neiderud 2015). These factors create favourable ecological conditions for the proliferation of urban populations of *Ae. aegypti* (Girard et al. 2020). For this reason, we chose to include human population densities of neighbourhoods in the analysis. Illiteracy was also considered, as education serves as the foundation for building a sustainable society, promoting cultural and social changes that enhance socio‐environmental improvement. It develops essential skills to strengthen individual and collective care for the environment, which is crucial for creating and maintaining mosquito reproduction‐free environments (Dias et al. 2022).

Spatio‐temporal analysis of the frequency of occurrence and distribution of breeding sites in cities such as Campo Grande can be very informative for dengue surveillance, considering its history as an epidemic center for dengue, characterised by high rates of incidence and morbidity (Espinosa et al. 2016; Jansen and Beebe 2010). By identifying and analysing these spatio‐temporal patterns, along with socio‐economic factors, we can gain more insight into the local infestation patterns of *Ae. aegypti*.

A study conducted in Maranhão, Brazil, identified that storage containers were the primary and most productive breeding sites for *Ae. aegypti* mosquitoes. The research indicated that larval productivity was notably higher during the rainy season, highlighting these containers' significant role in sustaining the mosquito population in Caxias (Soares‐da‐Silva et al. 2012).

Similarly, a study in Rio de Janeiro utilized satellite and street view imagery to identify *Ae. aegypti* breeding hotspots. It demonstrated a strong correlation between common breeding sites—such as water tanks, tires, plastic bags, and storm drains—and mosquito infestations. This information underscores important areas for targeted vector control efforts (Knoblauch et al. 2024).

Therefore, the aim of this study in Campo Grande was to verify whether the types of breeding sites changed seasonally over the course of 2 years (2022–2023). It was also intended to analyze whether socio‐economic factors such as neighbourhood population density and illiteracy rates impacted the frequency of occurrence of breeding site types. This understanding can be relevant for the development of more targeted and effective control strategies, such as public awareness campaigns, continuous surveillance, and monitoring of breeding sites, both by the government and the community.

## 2 Materials and Methods

### 2.1 Study Area

The study was conducted in Campo Grande, the capital of Mato Grosso do Sul, Brazil (20°28′13.40,737″S, 54°37′25.87,099″W) in 2022 and 2023. The city is located in the central region of the state with an area of 8.083 km^2^ and a population of 897.938 inhabitants, representing an urbanisation rate of 98.7% (Instituto Brasileiro de Geografia e Estatística 2025). According to the Brazilian Institute of Geography and Statistics/IBGE, in 2010, the city had an illiteracy rate of 5.0%, and by neighbourhood, it varies from 0.7% to 9.8%. The literacy rate is around 95% regardless of gender. In economic aspects, 64.7% of the population over 10 years of age is economically active. In Campo Grande, 92.3% of households live in a house, while the remaining live in apartments or other dwellings. On average, there are 3.1 residents per household (Sauer et al. 2012).

In Köppen's climate classification, the climate of Campo Grande falls within the transition zone between the humid mesothermal subtype (Cfa) and the humid tropical subtype (Aw). It is characterised by dry winters and rainy summers (Alvares et al. 2013; Custódio et al. 2019). The city is located within the neotropical phytogeographic region of the Cerrado (Agência Municipal de Meio Ambiente e Planejamento Urbano [PLANURB] 2016).

### 2.2 Data Collect

The Vector‐borne Disease Control Coordination (CCEV) acquired the data through a larval research surveillance program, and the information was recorded on worksheets. With those, it was possible to discriminate between each type of breeding site present in different urban residences. For LIRAa surveillance, cities are stratified, and in each stratum, blocks are evaluated by public epidemiology agents.

The worksheets were initially organised with year, month, epidemiological week, district, neighbourhood, activity, agent, address, property type, container code (A1, A2, B, C, D1, D2, E), containers (e.g., tire), number of tubes, and larvae stage. Below is a brief description of the survey approach. Houses were randomly selected for inspection throughout the city. These inspections were conducted by public epidemiology agents in the field. Upon encountering a larva, the agents proceeded to ascertain the specific type of breeding site and duly recorded this information on the provided paper forms. The larvae were stored in tubes and transported to CCEV for identification. The definitive species determination was subsequently entered into the spreadsheets. The initial classification of breeding site types was classified as in LIRAa (Silva et al. 2020). For the analysis, we reclassified by aggregating breeding site types that were categorised into subgroups (i.e., A and D breeding sites) as follows: water tank (A1 and A2), mobile container (B), fixed container (C), trash (D1 and D2), and natural breeding sites (E).

Based on the CCEV data, we reorganised the information where rows depict different neighbourhoods by month and year, as follows: neighbourhood, year, cycle, and breeding site type. While the original sheet did not have cycle information, we defined it as follows: Cycle 1 (January and February, weeks 1–9), cycle 2 (March and April, weeks 10–18), cycle 3 (May and June, weeks 19–22), cycle 4 (July and August, weeks 27–35), cycle 5 (September and October, weeks 36–43), and cycle 6 (November and December, weeks 44–52). The information provided by CCEV is missing data from cycles 1–3 in 2022 and cycle 2 in 2023. For each neighbourhood (sample unit of analysis), we gathered the socio‐economic covariates of population density and illiteracy rate.

These covariates can capture the spatiotemporal variance and prevalence of *Ae. aegypti* breeding sites. High human population density is associated with urbanised areas where closely packed housing and increased waste generation contribute to a greater availability of potential breeding habitats. In parallel, high illiteracy rates are often linked to lower levels of education, limited access to public health information, and inadequate sanitation infrastructure, all of which influence where and when breeding sites become more abundant (Lima et al. 2021; Souza et al. 2023).

### 2.3 Data Availability and Ethics Statement

Data are available upon request.

This study did not involve experimentation on humans or animals; therefore, approval by a Research Ethics Committee was not required.

### 2.4 Data Analysis

To compare the frequency of occurrence (defined as the number of breeding sites with *Ae. aegypti* larvae identified during the survey) of different breeding site types and how this frequency changes over the years, we developed a multilevel multinomial additive mixed model using a Bayesian approach. We used a multinomial distribution because the response variable (breeding site type) was composed of 5 categories (water tank, mobile container, fixed container, trash, and natural breeding site). The water tank was set as a reference level for coefficient estimation. We allowed a mixed structure because different neighbourhoods within the city were sampled repetitively over 2 years (2022 and 2023), six times per year. Therefore, we included a random intercept for each neighbourhood in each year, where years were nested within neighbourhoods. The additive structure was considered as a spline‐based smooth, with *k* = 3 dimensions, to verify how the proportion of occurrence of each breeding site type changes through the year (i.e., seasonality). The parameter *k* = 3 defines the number of basis functions (i.e., the degrees of freedom) used to smooth the temporal curve. This setting enables the model to detect broad, smooth changes in the occurrence of each breeding site type without overfitting short‐term fluctuations. Finally, because each neighbourhood has a different socio‐economic profile, the multilevel structure allowed us to test the effect of human population density and illiteracy rate on the importance of each breeding site type. The model was developed and run using the *brms* package in the R environment. We fit the model by running four 4000‐iteration chains, and checked for chains' mixing and normality of residuals.

## 3 Results

A total of 1716 breeding sites were surveyed, providing data on the types of containers identified: water tank (*n* = 336), mobile containers (*n* = 645), fixed containers (*n* = 153), trash (*n* = 574), and natural breeding sites (*n* = 8) throughout the city over the 2 years analysed (Table 1). The significance of the different container types is highlighted by their frequency in the surveys.

**TABLE 1:** Number of breeding sites throughout Campo Grande city in 2022–2023.

City region	Number of breeding sites									
	Water tank		Mobile containers		Fixed containers		Trash		Natural breeding site	
	2022	2023	2022	2023	2022	2023	2022	2023	2022	2023
Centro	7	14	14	41	2	8	7	36	0	1
Segredo	30	46	31	76	10	6	63	34	0	0
Prosa	4	16	0	12	4	10	4	26	0	1
Bandeira	12	26	26	74	14	12	25	53	0	1
Anhanduzinho	34	60	57	125	9	15	48	118	1	3
Lagoa	15	27	30	63	6	35	26	64	0	1
Imbirussu	17	28	34	62	9	13	25	45	0	0
**Total**	**119**	**217**	**192**	**453**	**54**	**99**	**198**	**376**	**1**	**7**

*Note:* Neighbourhoods by region: Centro (Centro, São Francisco, Cruzeiro, Jardim dos Estados, São Bento, Monte Líbano, Glória, Carvalho, Amambaí and Planalto); Segredo (José Abraão, Nasser, Seminário, Monte Castelo, Mata do Segredo, Coronel Antonino, Nova Lima); Prosa (Autonomista, Santa Fé, Carandá. Margarida, Novos Estados, Estrela Dalva, Veraneio, Noroeste); Bandeira (TV Morena, São Lourenço, Tiradentes, Maria Aparecida Pedrossian, Rita Vieira, Universitário, Moreninha); Anhanduzinho (Jockey Club, Piratinnga, Guanandi, Aero Rancho, Pioneiro, Alves Pereira, Centenário, Lageado, Los Angeles, Centro‐Oeste); Lagoa (Taveirópolis, Caiçara, União, Leblon, São Conrado, Tijuca, Caiobá, Batistão, Coophavila II, Tarumã); Imbirussu (Sobrinho, Santo Amaro, Santo Antônio, Panama, Popular, Nova CG, Núcleo Industrial).

There were several types of breeding sites per category, such as water tank (water tank, drum, barrel), mobile containers (plant pot, bucket, paint can, plastic container, grill, pet water bowl, plastic basin, coffee pot, compost pile, gallon, mobile water fountain, washing machine, plastic pool, plant, saucer, toy, cooking pot, can, glass jar with pepper, cooler box, soap holder, food can, tarp, meat box, pressure box, floor air cooler, plastic tray, basin, toilet bowl, tool box, paint tray, cart, cauldron, truck bed, loader shovel, chair, wheelbarrow, bottle cap, water jug, porcelain pot, baby bath tub, basket, watering can, sink, glass pot, religious item, trash lid, ground plate, teapot), fixed containers (pool, drain, floor aquarium, puddle, grease trap, ornamental fountain, drainage channel, concrete box, fixed laundry sink, fixed toilet bowl), trash—all items in this category were either unused or broken (plastic cup, tire, plastic container, paint can, tactile paving mould, ice cream tub, plastic pool, plastic bottle, discarded toilet bowl, concrete mixer, paint can lid, toy, trash can, cooking pot, scrap can, tarp, bucket, water can, cooler box, car scrap, water tank, plant pot, iron cooking pot, plastic bag, washing machine, margarine container, toilet cistern, tank, box, takeaway food container, refrigerator packaging, refrigerator stand, gallon jug, glass bottle, Skine bag, aluminium kettle, baby chair, old cabinet, beer can, Tupperware, TV tube, satellite dish, mug, yogurt container, coconut shell, helmet, pot lid), and natural breeding sites (aquatic plant, hollow tree trunk, bromeliad, unspecified plant).

**FIGURE 2:**
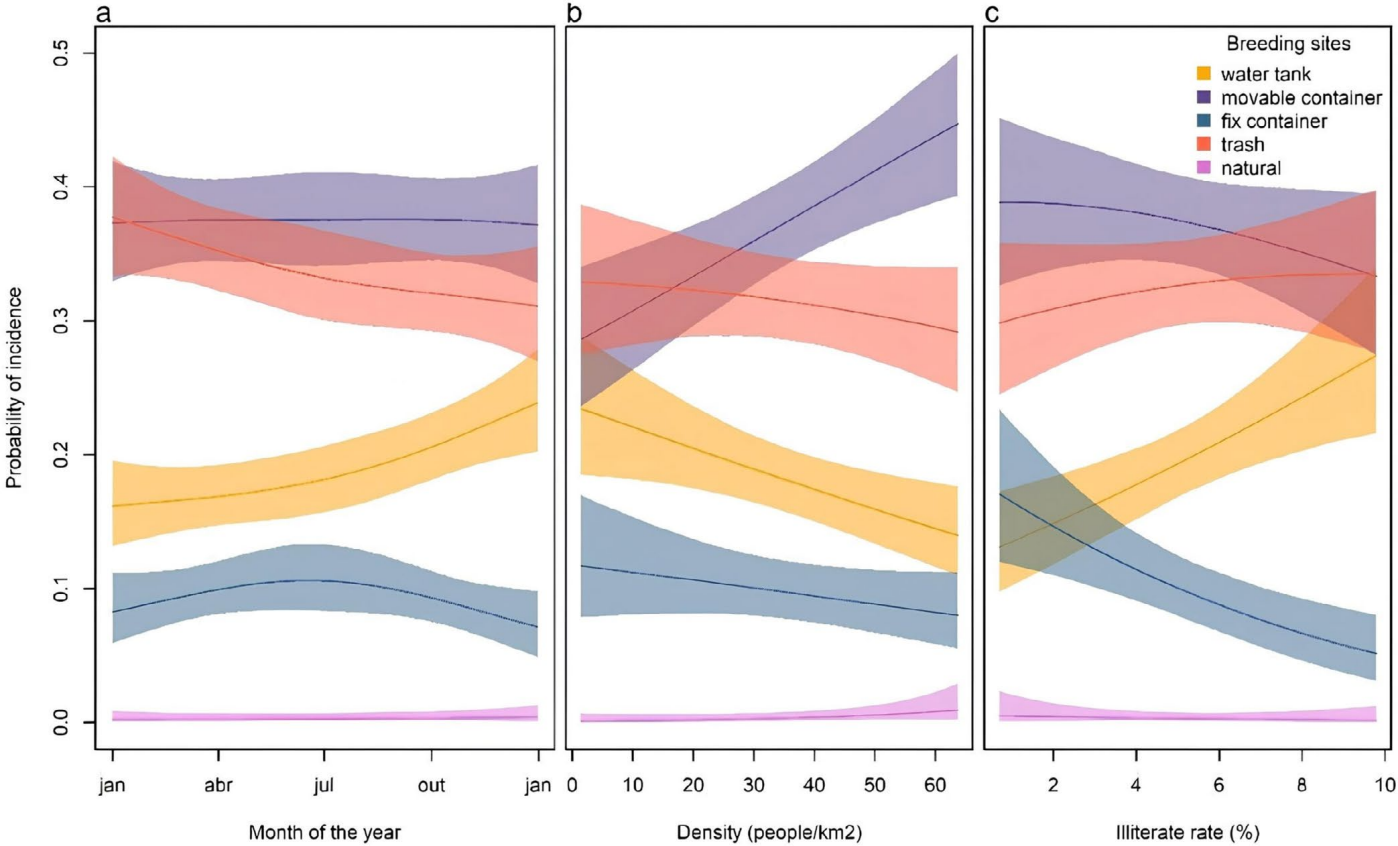
X‐axis: (a) Months of the year; (b) Population density per km^2^; (c) Illiteracy rate in percentage. Y‐axis: The probability of incidence of each breeding site. (a) Probability of incidence of breeding sites throughout the year. (b) Probability of incidence of breeding sites according to population density. (c) Probability of incidence of breeding sites according to illiterate rate.

Taking the water tank as reference breeding site type, trash (*β* = 0.64, IC95% = 0.16–1.12) and mobile containers (*β* = 0.61, IC95% = 0.13–1.09) were most frequently found breeding mosquitoes, while natural breeding sites much less (*β* = −4.52, IC95% = −7.42 to −2.11). Fixed containers functioning as mosquito breeding site were found as frequent as water tanks were (*β* = 0.14, IC95% = −0.49–0.79). Even though trash and mobile containers were the most frequently present throughout the year, we found seasonality in the frequency of these types of breeding sites (Figure 2a). Mobile containers had a subtle increase in frequency at the end of the year (*β* = 0.14, IC95% = 0.01–0.26), trash had a steep decrease (*β* = 0.20, IC95% = 0.07–0.33), while water tanks frequency increased. Despite its lower frequency, fixed containers showed a small increase in the middle of the year (*β* = 0.19, IC95% = 0.001–0.39); on the other hand, natural breeding sites were the least frequent and showed no seasonal signal (*β* = −0.06, IC95% = −0.81–0.63) (Figure 2a).

In terms of socio‐economic effects, overcrowded neighbourhoods showed a strong increase in mobile containers frequency (*β* = 0.02, IC95% = 0.01–0.02), and consequently a decrease in water tanks (Figure 2b). The number of infested fixed containers (*β* = 0.001, IC95% = −0.01–0.01), trash (*β* = 0.01, IC95% = −0.001–0.01), and natural breeding sites (*β* = 0.04, IC95% = −0.001–0.09) were not affected by population density. In terms of education, sites with higher illiterate rates showed a decreased importance of mobile containers (*β* = −0.10, IC95% = −0.16 to −0.03) and a strong increase in water tanks (Figure 2c). The frequency of fixed containers decreased (*β* = −0.21, IC95% = −0.31 to −0.12), and that of trash had a slight decrease (*β* = −0.07, IC95% = −0.14 to −0.001). The frequency of natural breeding sites (*β* = −0.19, IC95% = −0.58–0.18) was not affected by the illiteracy rate.

## 4 Discussion

Our study showed that mobile containers and trash have a higher probability of occurrence throughout the year, followed by water tanks and fixed containers. *Ae. aegypti* females exhibit an egg‐dispersal strategy known as “skip oviposition,” in which they distribute their eggs over multiple breeding sites. They also show a preference for artificial breeding sites over natural ones (Valle et al. 2021; Wilke et al. 2019). Our finding aligns with other studies, emphasizing the importance of artificial breeding sites for this vector (dos Santos Andrade et al. 2019; Maia et al. 2019; Valença et al. 2013).

Regarding the seasonality in the frequency of these types of breeding sites, mobile containers, such as plant saucers, vases, bottle caps, and trash such as old tires and garbage, have the highest probability of *Ae. aegypti* breeding (Martins et al. 2010; Morales‐Pérez et al. 2017; Yared et al. 2024; Rodrigues et al. 2023). These are followed by water tanks and fixed containers. Our results contrast with evidence from northeastern Brazil where water tanks were found to be the most predominant breeding sites (dos Santos Andrade et al. 2019). In our study, fixed containers showed a sharp increase in frequency in the middle of the year, coinciding with the dry season. This is likely due to the fact that these fixed containers—such as gutters, drains, and pools—can hold water that does not come from rain, and many are not properly cleaned. It has been observed that containers that are cleaned less frequently are four times more likely to become breeding sites for immature forms of *Ae. aegypti* (Overgaard et al. 2017). Overall, these breeding sites are critical to effective entomological control because *Ae. aegypti* eggs adhere to container walls, and any object that holds water can serve as a potential oviposition site (Reiter 2007). Therefore, it is critical to regularly clean and cover water tanks, gutters, drains, and any other containers that can hold water.

Areas with overcrowded neighbourhoods showed a higher probability of occurrence of larvae in mobile containers. Our findings show that the illiteracy rate and overcrowded neighbourhoods are significant socio‐economic factors that influence the spatio‐temporal reproductive dynamics of the local *Aedes* population. However, note that alternative socio‐economic variables such as household income, sanitation, and poverty have also been found to be correlated with the prevalence of breeding sites (Walker et al. 2011; da Silva and Scalize 2023; Guzmán and Kouri 2002; Albrieu‐Llinás et al. 2018; Obenauer et al. 2017).

*Aedes aegypti* is a well‐known mosquito species that thrives in human environments and depends mainly on man‐made containers for laying its eggs (Valle et al. 2021). High human population densities in densely populated urban areas create ideal ecological conditions that promote the growth of this mosquito population and increase the risk of disease outbreaks (Neiderud 2015). Our findings demonstrate that overcrowded neighbourhoods have a significantly higher occurrence of mobile containers that serve as breeding sites for mosquitoes. This supports similar observations made in Southeast Asia (Thammapalo et al. 2005). The author attributes this phenomenon to dense housing and spatial constraints, which contribute to the accumulation of discarded water‐holding containers. These containers create favourable microhabitats for the development of immature mosquitoes. These results highlight the important role of urban overcrowding as a factor that influences the spatial distribution and abundance of breeding habitats for *Ae. aegypti*.

Mobile containers and trash are more likely to be found in areas with higher illiteracy rates. Illiteracy, which is often linked to a lack of education, can affect the types of containers present in households (Quintero et al. 2009). This issue reflects insufficient basic literacy and numeracy skills and is associated with income inequality and negative life outcomes, including poverty (World Literacy Foundation 2023). It has been observed that households were more likely to have breeding sites if the head of the household had less than 6 years of primary school education (Morales‐Pérez et al. 2017), accumulating more containers and, in turn, offering more breeding sites for *Ae. aegypti* (Danis‐Lozano et al. 2002).

As already well documented, our study reinforced that natural breeding sites did not play a significant role for *Ae. aegypti* reproduction (Lima‐Camara et al. 2016; Santos et al. 2011; Mocellin et al. 2009). As previously highlighted, *Ae. aegypti* prefers artificial breeding sites, largely due to its association with human populations and the use of household containers (Lima‐Camara et al. 2016; Wilke et al. 2018). On the other hand, the use of natural breeding sites such as bromeliads can be relevant in certain localities, as observed in tropical areas of the USA. Even so, *Ae. aegypti* was the most abundant mosquito species found in ornamental bromeliads, suggesting that bromeliads may function as mosquito breeding sites (Wilke et al. 2018). However, it is important to note that in natural breeding sites, intraspecific competition between larvae and bacteria can lead to the absence of this vector (Santos et al. 2011).

Our findings provide valuable information to assist public epidemiology agents during vector control inspections. These insights can guide their efforts by highlighting specific types of containers that require more attention throughout the year. For instance, mobile containers and trash should be prioritized at the beginning of the year. From April to October, fixed containers should receive greater focus, and toward the end of the year, water tanks should be closely monitored. Nevertheless, it is important to emphasize that all types of containers must be evaluated regularly.

In overcrowded neighbourhoods, mobile containers should be prioritised. In contrast, in less crowded areas (0–20 people/km^2^), trash and water tanks are significant breeding sites. Areas with high illiteracy rates show a higher probability of mobile container incidence as breeding sites, providing public epidemiology agents with valuable guidance for targeted surveys. Our findings help to identify breeding sites with a higher likelihood of vector presence based on the neighbourhood's socio‐economic characteristics, enabling more effective inspection and elimination of containers, thereby contributing to epidemic prevention.

Our multilevel multinomial additive mixed model, developed using a Bayesian approach, enabled a comprehensive analysis of the probability of incidence of each *Ae. aegypti* breeding site type and its variation over time. The multinomial structure was well suited to the categorical nature of the response variable, encompassing five breeding site types. The mixed structure with random intercepts, combined with the spline‐based smooth additive structure, allowed the assessment of seasonal variations in the incidence of each breeding site type across time. Additionally, the multilevel model incorporated socio‐economic factors, enabling us to evaluate how external socio‐economic factors influenced the importance of each breeding site. In summary, our statistical approach offers a robust foundation for vector control modelling that integrates both socio‐economic and seasonal dynamics.

Entomological surveys provide essential data for assessing infestation rates, while socio‐economic factors help explain the underlying causes of vector proliferation. Both aspects should be taken into account for effective vector control (Dowling et al. 2013). This study improves the understanding of *Ae. aegypti* behaviour in an epidemic city by identifying breeding sites favourable for vector reproduction. The results also support health and education management strategies in the city, contributing to enhancing public awareness through targeted health education initiatives, such as promoting educational campaigns about each type of container and how to manage them to prevent them from becoming breeding sites.

## Conflicts of Interest

The authors declare no conflicts of interest.

## Data Availability

The data that support the findings of this study are available from the corresponding author upon reasonable request.
